# Revised Deep Learning Semiparametric Regression for Testing and Estimating Treatment Effect Heterogeneity in Observational Studies: A Lung Transplant Case Study

**DOI:** 10.64898/2026.04.13.718254

**Published:** 2026-04-18

**Authors:** Shuai Yuan, Fei Zou, Baiming Zou

**Affiliations:** Department of Biostatistics, University of North Carolina at Chapel Hill; Department of Biostatistics, University of North Carolina at Chapel Hill; Department of Biostatistics, University of North Carolina at Chapel Hill

**Keywords:** Causal inference, Heterogeneous treatment effects, Neural networks, Organ allocation, Precision medicine, Score test

## Abstract

Lung transplantation programs must decide when bilateral lung transplantation (BLT) offers meaningful functional benefit over single lung transplantation (SLT). Because donor and recipient characteristics jointly shape outcomes, the BLT-SLT contrast may differ across patients. However, analyzing observational registries poses a statistical challenge: apparent subgroup differences can be artifacts of complex confounding, while true heterogeneity can be missed or poorly quantified. Using a large national registry, we investigate whether the BLT effect varies across recipients and identify clinically relevant profiles of benefit using post-transplant lung function measured by forced expiratory volume in 1 second (FEV1).

We develop *deepHTL*, a framework that tests for treatment effect heterogeneity and estimates how the BLT-SLT effect varies with patient features. In extensive simulations designed to resemble registry-like confounding, *deepHTL* controls false positives for detecting heterogeneity and yields more accurate individualized effect estimates than common machine learning methods. In the lung transplant cohort, we find strong evidence of heterogeneity in the BLT-SLT effect on FEV1: younger, lower risk recipients with better baseline status show the largest FEV1 gains from BLT, whereas older, higher risk candidates exhibit diminished marginal benefit. These findings provide statistically grounded guidance for patient selection and allocation of scarce donor organs.

## 1 Introduction

Individualized decision-making is central to many areas of clinical practice, shifting the focus from the average treatment effect to identifying which patient subgroups a therapy benefits, has no meaningful effect on, or harms. Causal heterogeneous treatment effects (HTE) are essential for this precision medicine paradigm, as they characterize how intervention outcomes vary across patients with diverse demographic profiles, comorbidities, and biological markers. While HTE is well recognized in fields ranging from oncology to infectious disease (Piccart-Gebhart et al., 2005; Reck et al., 2016; RECOVERY Collaborative Group, 2021), the need for rigorous risk stratification is particularly acute in lung transplantation. In this setting, the choice between bilateral lung transplantation (BLT) and single lung transplantation (SLT) involves complex trade-offs that differ markedly across recipients characteristics. Factors such as age, body mass index (BMI), baseline frailty, and comorbidity burden can fundamentally alter the comparative effectiveness of BLT versus SLT on physiological outcomes like Forced Expiratory Volume in one second (FEV1) (Schaffer et al., 2015). Accurately testing and estimating HTE is critical for optimizing individual patient recovery and for effective allocation of scarce donor organs.

Inferring causal HTE from observational data, however, poses substantial analytical and modeling challenges. Unlike randomized trials, treatment assignment in observational registries is driven by complex, often interacting donor and recipient characteristics (e.g., age, sex, blood type), baseline disease severity, and comorbidity burden. This nonrandom assignment induces confounding that, if not rigorously addressed, can substantially bias treatment effect estimates (Rigdon et al., 2018). The problem is particularly acute in covariate-rich settings such as lung transplantation, where many donor and recipient variables may jointly influence both the transplant decision and post transplant outcomes through nonlinear, nonadditive pathways. In these regimes, conventional treatment assignment mechanism and outcome models can be severely misspecified and fail to capture key confounding structures. While the causal machine learning literature has rapidly expanded to offer flexible estimators for HTE, such as meta-learning strategies (e.g., T-, S-, and X-learners) (Künzel et al., 2019) which wrap off-the-shelf prediction algorithms, and Bayesian causal forests (Hahn et al., 2020; Wager and Athey, 2018) which provide powerful nonparametric modeling, these methods can be sensitive to hyperparameter tuning and prior specification (D’Amour et al., 2021; Curth and van der Schaar, 2021; Lu et al., 2017). Semiparametric regression based on Robinson’s transformation (Robinson, 1988) offers a robust alternative by applying Neyman-orthogonal estimating equations to mitigate the bias for estimating the nuisance functions (e.g., the conditional expectation of outcome estimation given observed covariates), a framework formalized by the quasi-oracle R-learner (Nie and Wager, 2021). Nevertheless, the robustness of the R-learner relies heavily on the quality of the nuisance estimators. In finite samples, conventional learners may fail to capture the complex confounding structures or overfit the data. Particularly, when the underlying treatment effect signal is strong, these learners are ineffective to deal with bimodal distributions even when HTE does not exist (Mi et al., 2019). Crucially, errors in these nuisance function estimations can propagate into the HTE estimates, leading to a systematic bias (Chernozhukov et al., 2018).

Furthermore, a fundamental yet often overlooked step is to separate genuine treatment effect heterogeneity from sampling variability. While recent deep learning approaches can outperform traditional learners for complex function approximations, the primary focus of this entire body of work remains on robust point estimation and interval construction of homogeneous treatment effect estimate (Mi et al., 2021). Without a statistically principled global test for the existence of HTE, investigators may overfit to noise, producing spurious subgroup recommendations and needlessly complex treatment rules. Formal hypothesis testing for the existence of treatment effect heterogeneity is largely overlooked, with existing tests restricted to randomized trials (Chang et al., 2021) or prespecified subgroups (Dai and Stern, 2022). Consequently, there remains a lack of an integrated framework that can simultaneously deliver efficient and robust HTE estimation along with a calibrated global test for its presence in observational studies.

Motivated by these gaps and the critical need for reliable evidence in high-stakes clinical decision-making, we propose a unified framework, *deep Heterogeneous Treatment Learning* (*deepHTL*). This framework integrates three key strategies to robustly test for and estimate causal HTE in observational studies. First, we employ bagged deep neural networks (bagged-DNN) to estimate nuisance functions, ensuring robust and accurate estimations while mitigating stability issue in traditional DNN models under the finite sample setting (Mi et al., 2021; Zou et al., 2025). Second, we estimate heterogeneous treatment effects via a semiparametric regression using the revised nuisance function estimation, a step designed to reduce bias that often undermines standard R-learners. Third, to rigorously assess the existence of treatment effect heterogeneity, we implement a global kernel score test and calculate its significance using the Davies method (Liu et al., 2007; Davies, 1980), complemented by a cross-fitted permutation test to ensure robust and principled inference under finite sample settings.

We evaluate the performance of *deepHTL* through extensive simulation studies, showing improved estimation accuracy relative to standard R-learners and demonstrating that the proposed testing methods maintain nominal Type I error while achieving high power in detecting treatment effect heterogeneity. We then apply *deepHTL* to a large cohort from the United Network for Organ Sharing (UNOS) registry to investigate the comparative effectiveness of BLT vs. SLT on post-transplant lung function, measured by FEV1. The analysis reveals statistically significant and clinically meaningful heterogeneity: younger recipients with lower baseline risk experience the largest functional gains from BLT, whereas older, higher risk patients exhibit attenuated marginal benefit. These results illustrate how *deepHTL* can support more targeted decision-making and potentially refine allocation criteria for scarce donor organs. Although we focus on lung transplantation, the framework is broadly applicable to other observational settings where individualized treatment effects are of primary interest.

The remainder of the paper is organized as follows. Section 2 describes the detailed framework of *deepHTL*. In Section 3, we report extensive results from simulation studies mimicing various practical confounding and HTE settings. Section 4 presents the UNOS case study application, and we conclude the paper in Section 5 with a brief discussion.

## 2 Methods

To model potentially complex heterogeneous treatment effects and accommodate intricate confounding structures commonly observed in clinical practice, we consider the following semiparametric regression model:

$$Y_{i}=\tau \left(X_{i}\right)Z_{i}+f\left(X_{i}\right)+\varepsilon_{i}$$

where $Y_{i}\in R$ denotes the observed outcome, $Z_{i}\in \{0,\;1\}$ is the binary treatment assignment indicator, and $X_{i}\in R^{p}$ represents the set of vector of pre-treatment covariates for the $i^{th}$ individual (i=1,…,n). The random error term $\varepsilon_{i}$ is assumed to satisfy $E\left[\varepsilon_{i}|X_{i},Z_{i}\right]=0$ with a finite variance. To accommodate potential complex confounding structures, we leave the nuisance function f(X) unspecified. Similarly, the treatment effect of interest, τ(X), is defined as an unspecified function of X, allowing for flexible modeling of treatment effect heterogeneity. Under this semiparametric regression framework, we present a set of robust and powerful methods to estimate and test for the existence of treatment effect heterogeneity as described in the following subsections.

### 2.1 Deep Heterogeneous Treatment Learning for Estimating HTE

To avoid estimating the unknown nuisance function f(X) in the above semiparametric regression model, we employ the Robinson transformation:

(2.1) $$Y-\mu \left(X\right)=\tau \left(X\right)\left\{Z-e\left(X\right)\right\}+\varepsilon ,$$

where μ(X)=E[Y∣X] and e(X)=E[Z∣X] represent nuisance functions that can be pre-estimated straightforwardly using, for example, the kernel methods. (Robinson, 1988). Alternatively, many standard machine learning methods such as Lasso (Tibshirani, 1996), XGBoost (Chen and Guestrin, 2016), and Kernel Ridge Regression (KRR) (Vovk, 2013), as utilized by Nie and Wager (2021) can be adopted for estimating these nuisance functions. However, they often struggle in the presence of complex, for example, nonlinear and nonadditive confounding. In such scenarios, nuisance estimators trained on finite samples using these machine learning models can exhibit systematic bias, and such errors propagate into the estimation of τ(X) (Chernozhukov et al., 2018) since they may not accurately approximate the potential complex associations in these scenarios. While deep neural network (DNN) can universally and accurately approximate complex functions asymptotically (Hornik et al., 1989; Cybenko, 1989), its performance under finite sample settings can be poor where a DNN model may not converge to the global optimal due to random parameter initialization. Bagged-DNN method addresses this challenge by aggregating predictions from multiple bootstrap-trained DNN models while explicitly discarding poorly performed DNN models, thereby stabilizing the fits against the randomness of network initialization. Based on this observation, to obtain more reliable nuisance function estimates, we implement a bagged-DNN model enhanced with a filtering mechanism (Mi et al., 2019, 2021; Zou et al., 2025).

To further mitigate overfitting and induce the orthogonality of nuisance parameter estimation errors, we randomly partition the observation indices {1,…,n} into K disjoint folds, denoted by $I_{1},\ldots ,I_{K}$. For each fold k, the bagged-DNN models for μ(X) and e(X) are trained on the out-of-fold samples to generate predictions exclusively for the held-out observations $i\in I_{k}$. We then use these out-of-sample nuisance estimates $\overset{ˆ}{\mu }\left(X_{i}\right)$ and $\overset{ˆ}{e}\left(X_{i}\right)$ to estimate the heterogeneous treatment effects by minimizing the squared loss derived from (2.1):

(2.2) $$\overset{ˆ}{\tau }(\cdot )=arg\;\underset{\tau }{min}\frac{1}{n}\sum_{i=1}^{n}{\left\{(Y_{i}-\overset{ˆ}{\mu }\left(X_{i}\right))-\tau \left(X_{i}\right)\left(Z_{i}-\overset{ˆ}{e}\left(X_{i}\right)\right)\right\}}^{2},$$

and minimization is performed using the bagged-DNN. However, in settings like transplantation where the treatment effect magnitude |τ(X)| can be large, the finite sample accuracy of this estimator can deteriorate. As shown in (2.1), the residual Y-μ(X) comprises τ(X){Z-e(X)} and random noise ε. When |τ(X)| is small, the residual is dominated by ε, which typically follows a standard unimodal distribution (e.g., Gaussian) that is efficiently learned by mean-squared-error minimization. Conversely, when |τ(X)| is large, the dominance of τ(X){Z-e(X)} induces residual bimodality, which introduces estimation bias in $\overset{ˆ}{\mu }(X)$ and propagates to $\overset{ˆ}{\tau }(X)$.

To address this, we decompose the treatment effect into a constant average component and a residual heterogeneous component: $\tau (X)=\tau_{0}+r(X)$. We reformulate (2.1) with the following model:

(2.3) $$Y^{*}-E[Y^{*}|X]=r\left(X\right)\left\{Z-e\left(X\right)\right\}+\varepsilon ,$$

where $Y^{*}=Y-\tau_{0}Z$ denotes the centered outcome and $\mu^{*}(X)=E(Y^{*}|X)$ is estimated using the bagged-DNN via the same cross-fitting procedure. This centering step effectively removes the large main effect of treatment and strengthens the unimodal residual structure, allowing $\mu^{*}(X)$ to be estimated more accurately. We estimate the constant average component $\tau_{0}$ via a computationally efficient double robust estimator:

(2.4) $${\overset{ˆ}{\tau }}_{0}=\frac{\sum_{i=1}^{n}\left\{Y_{i}\;-\;\overset{ˆ}{\mu }\left(X_{i}\right)\right\}\left\{Z_{i}\;-\;\overset{ˆ}{e}\left(X_{i}\right)\right\}}{\sum_{i=1}^{n}{\left\{Z_{i}\;-\;\overset{ˆ}{e}\left(X_{i}\right)\right\}}^{2}}.$$

The residual heterogeneous component estimator $\overset{ˆ}{r}(X)$ is then obtained by minimizing the squared loss:

(2.5) $$\overset{ˆ}{r}(\cdot )=arg\;\underset{r}{min}\frac{1}{n}\sum_{i=1}^{n}{\left\{\left(Y_{i}-{\overset{ˆ}{\tau }}_{0}Z_{i}-{\overset{ˆ}{\mu }}^{*}\left(X_{i}\right)\right)-r\left(X_{i}\right)(Z_{i}-\overset{ˆ}{e}\left(X_{i}\right))\right\}}^{2}.$$

Combining the constant and residual components produces the revised estimator of HTE:

(2.6) $${\overset{ˆ}{\tau }}^{*}(X)={\overset{ˆ}{\tau }}_{0}+\overset{ˆ}{r}(X).$$

A complete HTE estimation procedure based on *deepHTL* is summarized in Algorithm 1.

**Algorithm 1:** **deepHTL:** Estimating Heterogeneous Treatment Effects

1:	**Input:** Observations ${\left\{\left(Y_{i},Z_{i},X_{i}\right)\right\}}_{i=1}^{n}$ partitioned into K folds.
2:	**for** each fold k=1,…,K do
3:	Estimate nuisance functions $\overset{ˆ}{e}\left(X_{i}\right)$ and $\overset{ˆ}{\mu }\left(X_{i}\right)$ for $i\in I_{k}$.
4:	**end for**
5:	Compute the constant effect ${\overset{ˆ}{\tau }}_{0}$ using (2.4) and the centered outcome $Y_{i}^{*}=Y_{i}-{\overset{ˆ}{\tau }}_{0}Z_{i}$.
6:	**for** each fold k=1,…,K **do**
7:	Estimate the centered nuisance function ${\overset{ˆ}{\mu }}^{*}\left(X_{i}\right)=E[Y_{i}^{*}|X_{i}]$ for $i\in I_{k}$.
8:	**end for**
9:	Estimate residual heterogeneity $\overset{ˆ}{r}(X)$ by minimizing the squared loss function in (2.5)
10:	**Output:** Revised HTE estimate ${\overset{ˆ}{\tau }}^{*}(X)={\overset{ˆ}{\tau }}_{0}+\overset{ˆ}{r}(X)$.
**Note:** All functions are estimated using the bagged-DNN.	

### 2.2 Testing for the Existence of HTE

In clinical practice, it is critically important to test the existence of HTE to avoid deploying unnecessarily complex individualized treatment rules. In observational registries, searching for subgroups without a global protection against Type I error risks identifying “false positive” heterogeneity driven by noise or residual confounding. We therefore test the null hypothesis $H_{0}:Var\{\tau (X)\}=0$, which implies that τ(X) reduces to an unknown constant under the null. Specifically, we propose two procedures for testing the existence of HTE: a computationally efficient analytic method using the kernel score test, and a more robust, albeit computationally intensive, empirical method based on a permutation procedure as described in the following.

#### Kernel Score Test:

Accurately estimating the constant effect is essential for constructing residuals that capture genuine departures from the null hypothesis rather than artifacts of estimation error. Although ${\overset{ˆ}{\tau }}_{0}$ in (2.4) provides an initial (“unrevised”) estimate, its performance can deteriorate in finite samples when the treatment effect is large in magnitude, i.e., the non-Gaussian term may dominate, leading to inaccurate estimation of $\overset{ˆ}{\mu }(X)$. To address this issue while preserving our cross-fitting architecture for the nuisance parameters, we incorporate a bias correction step. Specifically, we compute a global revised estimator, denoted as ${\overset{ˆ}{\tau }}^{*}$:

(2.7) $${\overset{ˆ}{\tau }}^{*}={\overset{ˆ}{\tau }}_{0}+\frac{\sum_{i=1}^{n}\left(Y_{i}\;-\;{\overset{ˆ}{\tau }}_{0}Z_{i}\;-\;{\overset{ˆ}{\mu }}^{*}\left(X_{i}\right)\right)\left(Z_{i}\;-\;\overset{ˆ}{e}\left(X_{i}\right)\right)}{\sum_{i=1}^{n}{\left(Z_{i}\;-\;\overset{ˆ}{e}\left(X_{i}\right)\right)}^{2}}.$$

Using ${\overset{ˆ}{\tau }}^{*}$, we derive the raw residual score $u_{i}$ and its studentized version $s_{i}$ for each observation i=1,…,n:

$$u_{i}=\frac{Y_{i}\;-\;{\overset{ˆ}{\tau }}_{0}Z_{i}\;-\;{\overset{ˆ}{\mu }}^{*}\left(X_{i}\right)}{Z_{i}\;-\;\overset{ˆ}{e}\left(X_{i}\right)}-({\overset{ˆ}{\tau }}^{*}-{\overset{ˆ}{\tau }}_{0}),\;s_{i}=\frac{\sqrt{w_{i}}u_{i}}{\overset{ˆ}{\sigma }},$$

where $w_{i}={\left(Z_{i}-\overset{ˆ}{e}\left(X_{i}\right)\right)}^{2}$ and ${\overset{ˆ}{\sigma }}^{2}\approx n^{-1}\sum_{i=1}^{n}w_{i}u_{i}^{2}$ estimates the conditional variance $Var\left(\varepsilon_{i}|X_{i},Z_{i}\right)$. Under the null hypothesis $H_{0}$ and mild regularity conditions, using cross-fitted nuisance parameters allows the estimation error to be asymptotically orthogonal to the score (Chernozhukov et al., 2018). Thus, the raw residual admits the asymptotic expansion $u_{i}\approx \frac{\varepsilon_{i}}{Z_{i}-e\left(X_{i}\right)}+o_{p}(1)$, the leading term of $u_{i}$ is proportional to the noise. Consequently, the studentized scores $s_{i}=\frac{\sqrt{w_{i}}u_{i}}{\overset{ˆ}{\sigma }}$ are marginally asymptotically standard normal.

Let $s={\left(s_{1},\ldots ,s_{n}\right)}^{⊤}$ and $a={\left(\sqrt{w_{1}},\ldots ,\sqrt{w_{n}}\right)}^{⊤}$. By definition of the weighted least squares objective used to estimate the constant treatment effect, the resulting residuals satisfy $a^{⊤}s=0$. This linear constraint inherently induces dependence among the components of s. To properly accommodate this structural restriction, we project s onto the orthogonal complement of span(a):

(2.8) $$\tilde{s}=Ps,\;P=I_{n}-\frac{aa^{⊤}}{a^{⊤}a}=I_{n}-\frac{\sqrt{w}{\sqrt{w}}^{⊤}}{\sum_{i=1}^{n}w_{i}}.$$

Here P is symmetric and idempotent ($P^{⊤}=P,P^{2}=P$), satisfies Pa=0, and has rank n-1 provided a≠0. Following Liu et al. (2007), we construct the quadratic form statistic $Q={\tilde{s}}^{⊤}K\tilde{s}$, where K is a symmetric positive semidefinite kernel matrix with entries $K_{ij}=K\left(X_{i},X_{j}\right)$ for some Mercer kernel K(⋅,⋅) (e.g., Gaussian RBF). Under the null hypothesis and $s\overset{d}{\approx }N(0,\Sigma )$, the quadratic form admits a weighted chi-square representation:

$$Q\overset{d\;}{\to }\sum_{j=1}^{r}\lambda_{j}\chi_{1,j}^{2},$$

where ${\left\{\lambda_{j}\right\}}_{j=1}^{r}$ are the nonzero eigenvalues of PKP (with rank r≤n-1) and $\chi_{1,j}^{2}$ are independent central chi-square random variables with 1 degree of freedom. An analytic p-value is obtained using the Davies method (Davies, 1980):

$$p=Pr\left(\sum_{j=1}^{r}\lambda_{j}\chi_{1,j}^{2}\geq Q\right).$$

#### Permutation Test:

While the kernel score test provides a powerful and computationally efficient analytic framework, its validity relies on asymptotic convergence to a mixture of chi-square distributions. The projection matrix P corrects for the degree of freedom lost in estimating the global constant treatment effect, but it may not fully account for the effective degrees of freedom consumed by the complex DNN nuisance estimators. In challenging finite sample settings (e.g., high dimensionality, high noise, moderate sample sizes), this uncaptured estimation bias may compromise the asymptotic approximation and lead to mild Type I error inflation. To provide a more reliable and robust inference tool for testing existence of HTE, we propose a permutation test. By empirically constructing the null distribution, this approach aims to naturally accommodate the variance structure and control Type I error without relying strictly on asymptotic assumptions.

We construct a test statistic based directly on the out-of-sample prediction loss. Using the same K disjoint folds $I_{1},\ldots ,I_{K}$ from the nuisance estimation stage, we cross-fit the treatment effect model in (2.6). For each fold k∈{1,…,K}, the model is trained exclusively on the remaining K-1 folds to generate predictions ${\overset{ˆ}{\tau }}^{*}\left(X_{i}\right)$ for the holdout observations $i\in I_{k}$. The observed mean squared error (MSE) across all K folds is calculated as:

(2.9) $$L_{obs}=\frac{1}{n}\sum_{k=1}^{K}\sum_{i\in I_{k}}{\left\{\left(Y_{i}^{*}-{\overset{ˆ}{\mu }}^{*}\left(X_{i}\right)\right)-{\overset{ˆ}{\tau }}^{*}\left(X_{i}\right)(Z_{i}-\overset{ˆ}{e}\left(X_{i}\right))\right\}}^{2}.$$

To generate the null distribution, we perform B random permutations. For each permutation b=1,…,B, we operate within each fold k∈{1,…,K} to randomly shuffle the indices of the holdout predictions within their respective treatment arms ($Z_{i}\in \{0,\;1\}$). Letting $\pi^{\left(b\right)}(i)$ denote the permuted index for observation i, the permuted loss is:

(2.10) $$L_{perm}^{\left(b\right)}=\frac{1}{n}\sum_{k=1}^{K}\sum_{i\in I_{k}}{\left\{\left(Y_{i}^{*}-{\overset{ˆ}{\mu }}^{*}\left(X_{i}\right)\right)-{\overset{ˆ}{\tau }}^{*}(X_{\pi^{\left(b\right)}(i)})(Z_{i}-\overset{ˆ}{e}\left(X_{i}\right))\right\}}^{2}.$$

The empirical *p*-value is computed as the proportion of permutations where the permuted loss is less than or equal to the observed loss:

$$p_{perm}=\frac{1}{B\;+\;1}\left(1+\sum_{b=1}^{B}I\left\{L_{perm}^{\left(b\right)}\leq L_{obs}\right\}\right).$$

Under the null hypothesis, the covariates X provide no informative signal for treatment heterogeneity. Consequently, breaking the alignment between the observed data and the predicted heterogeneity ${\overset{ˆ}{\tau }}^{*}\left(X_{i}\right)$ via permutation does not systematically alter the loss, meaning $L_{\text{obs}}$ and $L_{\text{perm}\;}^{\left(b\right)}$ are drawn from the same underlying distribution. Under the alternative hypothesis $H_{1}$, shuffling destroys the alignment with the true heterogeneous signal, causing $L_{\text{perm}\;}^{\left(b\right)}$ to be larger than $L_{\text{obs}}$.

This framework applies identically to the unrevised estimator $\overset{ˆ}{\tau }\left(X_{i}\right)$ by cross-fitting the loss function in (2.2). The complete procedure for testing the existence of HTE is summarized in Algorithm 2.

**Algorithm 2:** **deepHTL:** Testing for Heterogeneous Treatment Effects

1:	**Input:** Observations ${\left\{\left(Y_{i},Z_{i},X_{i}\right)\right\}}_{i=1}^{n}$, cross-fitting folds K, permutations B.
2:	**Kernel Score Test**
3:	Partition data into K disjoint folds.
4:	Obtain out-of-fold nuisance estimates $\overset{ˆ}{e}\left(X_{i}\right)$, $\overset{ˆ}{\mu }\left(X_{i}\right)$, and ${\overset{ˆ}{\mu }}^{*}\left(X_{i}\right)$ for all i.
5:	Compute the revised constant estimator ${\overset{ˆ}{\tau }}^{*}$ via (2.7).
6:	Compute studentized residuals s and the projection matrix s and the projection matrix P in (2.8).
7:	Calculate test statistic $Q=s^{⊤}PKPs$ and derive $p_{\text{davies}}$.
8:	**Permutation Test**
9:	Train the target model (2.2) and (2.6) via cross-fitting.
10:	Obtain out-of-fold predictions $\overset{ˆ}{\tau }\left(X_{i}\right)$ for the holdout fold k.
11:	Calculate the observed loss $L_{\text{obs}}$ via (2.9).
12:	**for** b=1,…,B **do**
13:	Permute treatment indices within folds and calculate $L_{\text{perm}\;}^{\left(b\right)}$ via (2.10).
14:	**end for**
15:	Compute $p_{\text{perm}}=\frac{1}{B+1}\left(1+\sum_{b=1}^{B}I\left\{L_{\text{perm}\;}^{\left(b\right)}\leq L_{\text{obs}}\right\}\right)$.
16:	**Output:** $p_{\text{davies}}$ and $p_{\text{perm}}$.
**Note:** All functions are estimated using the bagged-DNN.	

## 3 Simulation Studies

We evaluate the finite sample performance of *deepHTL* through extensive simulation studies designed to mimic the complex confounding structures commonly encountered in observational biomedical registries. Our primary objectives are to assess (i) the performance of the proposed kernel score and permutation tests under the null hypothesis $H_{0}:Var\{\tau (X)\}=0$, (ii) the power of the test to detect heterogeneity under the alternative hypothesis $H_{1}:Var\{\tau (X)\}>0$, and (iii) the estimation accuracy of the proposed HTE estimator across diverse settings. To this end, we generate data from the following outcome model:

$$Y_{i}=\tau \left(X_{i}\right)Z_{i}+f\left(X_{i}\right)+\varepsilon_{i},\;i=1,\ldots ,n,$$

where $X_{i}∼{𝒩}_{p}\left(0,I_{p}\right)$ denotes the *p*-dimensional covariate vector (including confounders and nuisance factors), and $\varepsilon_{i}∼𝒩\left(0,\sigma^{2}\right)$ is an independent noise term. Binary treatment assignment mechanism follows the following:

$$Z_{i}|X_{i}∼Bernoulli\left\{e\left(X_{i}\right)\right\},\;e(X)={logit}^{-1}\left\{0.8\;sin\left(\pi X_{1}X_{2}\right)+0.6\;X_{3}X_{4}+0.5\;tanh\left(X_{5}\right)\right\}.$$

The structural nuisance function is set to $f(X)=log\left(\left|X_{1}\right|+1\right)-X_{2}^{2}+sin\left(X_{3}\right)+0.5X_{4}X_{5}$, and the treatment effect function τ(X) varies by experiment as the following:

$$\tau (X)=\left\{\begin{array}{ll} 0, & (\text{Zero}\;\text{effect}\;\text{without}\;\text{HTE}),\\ 3, & (\text{Large}\;\text{effect}\;\text{without}\;\text{HTE}),\\ -1+2\;log\left(exp\left(\sum_{j=1}^{5}X_{j}\right)+1\right), & (\text{Simple}\;\text{HTE}),\\ -1+X_{1}X_{2}+{cos}^{2}\left(X_{3}\right)+max\left(X_{4}-X_{5},0\right) & (\text{Complex}\;\text{HTE}). \end{array}\right.$$

We evaluate performance over a grid of sample sizes n∈{1000,2000}, covariate dimensions p∈{20,40}, and noise levels σ∈{1,3}, using 1000 independent replicates per configuration.

### 3.1 Performance on Testing HTE

We first evaluate the performance of the proposed testing procedures under the null hypothesis of homogeneous treatment effects. To probe robustness across effect magnitudes, we consider two homogeneous scenarios: a zero effect (τ=0) and a large constant effect (τ=3). For each scenario, we construct test statistics using both the unrevised estimator $\overset{ˆ}{\tau }$ and the revised estimator ${\overset{ˆ}{\tau }}^{*}$. We report the empirical Type I error rates across 1,000 replicates at the nominal level α=0.05 for the kernel score test and the permutation test (with B=2000 permutations).

Table 1 shows that the revised estimator delivers uniformly reliable Type I error control across all configurations, whereas the unrevised estimator can break down sharply when the treatment signal is strong relative to noise. Under τ=0 using the kernel score test, both tests constructed from the revised and unrevised estimators generally produce type I errors that are generally close to nominal, with only modest inflation in the most challenging high-dimensional/high-noise regime (n=1000,p=40,σ=3). In contrast, under τ=3, the unrevised estimator exhibits substantial and systematic inflation whenever the signal-to-noise ratio is high (notably σ=1), with rejection rates ranging from 0.15 to 0.30 (e.g., 0.299 at n=2000,p=20,σ=1), far exceeding the 0.05 target. This inflation is consistent with the mechanism described in Section 2.2: when τ is large, the residual Y-μ(X) is dominated by τ{Z-e(X)} rather than ε, producing a pronounced bimodal structure. As a result, the nuisance function estimator $\overset{ˆ}{\mu }(X)$ becomes inaccurate, which in turn biases the treatment effect estimate ${\overset{ˆ}{\tau }}_{0}$. Moreover, the residual score computed using this unrevised estimate ${\overset{ˆ}{\tau }}_{0}$ can deviate substantially from unimodality and Gaussianity, leading to uncontrolled Type I error.

This failure mode is eliminated by the proposed revision. Across every setting in Table 1 including the low noise, large effect regimes where the unrevised method is most anti-conservative, the revised estimator ${\overset{ˆ}{\tau }}_{0}^{*}$ yields near-nominal Type I error (typically 0.04–0.06), demonstrating robustness to sample size, dimensionality, and signal-to-noise ratio. Moreover, the remaining mild inflation in the hardest regimes (p=40,σ=3) attenuates as n increases (to ≈ 0.06 at n=2000), consistent with improved nuisance function estimation in larger samples typical of registry studies (e.g., our UNOS application with n>10,000). Furthermore, the permutation test successfully relieves the mild inflation observed in the hardest analytical settings (e.g., reducing the rejection rate from 0.085 to roughly 0.06 at n=1000,p=40,σ=3). Because the permutation test relies on random shuffling rather than asymptotic approximations, it avoids relying on the asymptotic normality of the residuals, providing tighter empirical control when conditions are challenging.

Although strictly controlling Type I error is paramount to preventing false discoveries, a practical testing procedure must also demonstrate sufficient power to uncover true heterogeneity when it exists. We maintain the data generating process from the previous section but introduce covariate-dependent treatment effects τ(X) under two different scenarios. **Scenario I** posits a simple, mildly nonlinear effect structure, $\tau (X)=-1+2\;log\left(exp\left(\sum_{j=1}^{5}X_{j}\right)+1\right)$, while **Scenario II** introduces complex nonlinear interactions, $\tau (X)=-1+X_{1}X_{2}+{cos}^{2}\left(X_{3}\right)+max\left(X_{4}-X_{5},0\right)$. Having established that the unrevised approach fails to control the Type I error rate, we restrict our power evaluations to the properly calibrated revised estimator. For each setting, we report the empirical power, defined as the proportion of replicates where the null hypothesis is rejected at the nominal significance level α=0.05.

In **Scenario I**, the treatment effect function is simple, and the heterogeneity signal is pronounced (average Var{τ(X)}≈6.05). Consequently, detection is straightforward: both estimators under the two testing frameworks achieve a power of 1.0 across all considered sample sizes and noise levels (results not tabulated). This confirms that the proposed testing methods reliably identify overt heterogeneity without loss of sensitivity.

**Scenario II** presents a more challenging test bed due to its lower signal variance and complex functional form. Due to its inflated Type I error (Table 1), we omit the unrevised test from our power evaluation. Table 2 reports results exclusively for the proposed revised estimator. Similar to the Type I error analysis, statistical power is sensitive to the data complexity. Since detecting heterogeneity relies on distinguishing systematic variation in τ(X) from random error, increasing the noise level σ or the dimension p naturally attenuates power by diluting the signal-to-noise ratio. The test demonstrates a strong responsiveness to sample size. Under the most challenging scenario (σ=3,p=40), increasing n from 1000 to 2000 more than doubles the analytical power (43.7% to 89.3%). The permutation test achieved a similar power pattern to the kernel score test, confirming that both frameworks reliably detect the heterogeneity signal in larger samples. These results confirm the revised estimator balances rigorous error control with the sensitivity needed for practical observational studies.

### 3.2 Performance on Estimating HTE

Having established the validity of the testing procedure, we next evaluate the accuracy of HTE estimation. We maintain the data-generating process and the two heterogeneous scenarios from the previous section and apply the HTE estimator in Section 2.1. For each configuration in the simulation grid, models are trained on a dataset and evaluated on an independent test set of equal size using the logarithmic mean squared error (log-MSE).

To benchmark the proposed *deepHTL* framework, we compare it with three machine learning methods: Least absolute shrinkage and selection operator (*Lasso* (Tibshirani, 1996)), eXtreme Gradient Boosting (*XGBoost* (Chen and Guestrin, 2016)), and Kernel Ridge Regression (*KRR* (Vovk, 2013)). Each learner is used consistently throughout the estimation pipeline to obtain the estimation of the nuisance functions μ(X) and e(X), as well as the treatment effect function τ(X). To isolate the contribution of our revision step, we implement two versions of every learner: the unrevised estimator in (2.2) (denoted ***R–learner***, e.g., *R–Lasso*) and the proposed revised estimator in (2.6)(denoted ***Rev–learner***, e.g., *Rev–XGBoost*). Within this comparison framework, *deepHTL* corresponds to the revised estimator implemented with bagged-DNN, while its unrevised counterpart is denoted as *R–DNN*.

**Figure 1:**
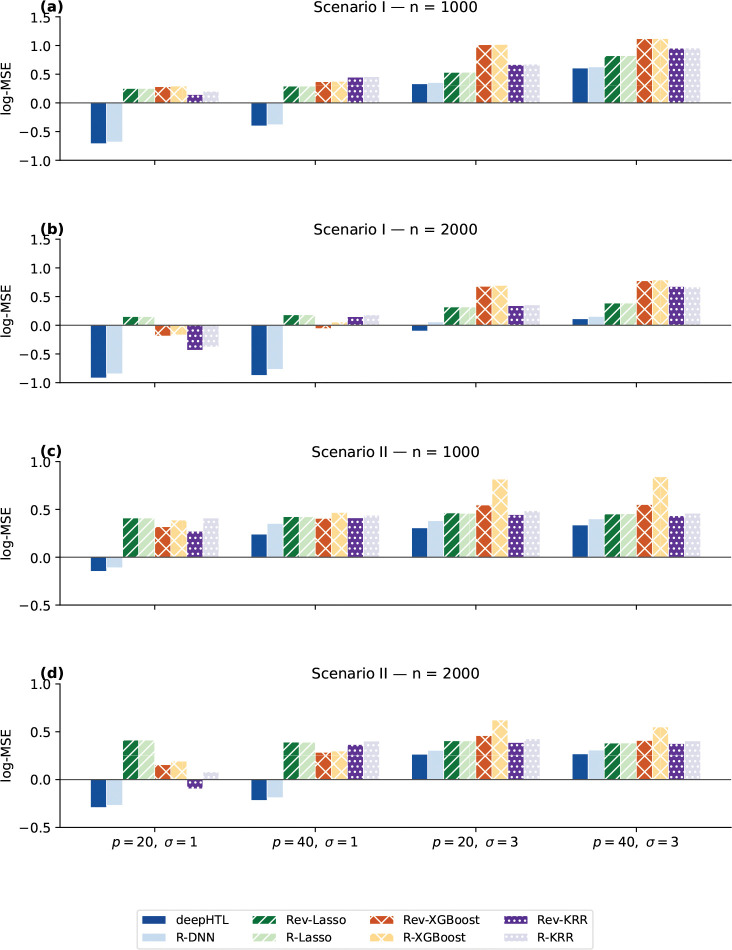
Log-MSE of $\overset{ˆ}{\tau }(X)$ across simulation settings for all HTE estimators.

Figure 1 summarizes the predictive performance (log-MSE) across simulation settings. The results highlight a critical interaction between learner capacity and the utility of the revision step. For parametric methods like Lasso, the revised estimator offers negligible benefit, as the linear additive assumption fails to capture the complex nuisance structure μ(X). In contrast, flexible learners (XGBoost, KRR, DNN) exhibit substantial gains in **Scenario II**. For example, the revised estimator reduces the error metric by 0.1 ~ 0.2 for both XGBoost and KRR. Similarly, *deepHTL* consistently improves, lowering the log-MSE by over 0.19 in complex configurations (e.g., n=1000, p=40, σ=1). In the smoother **Scenario I**, benefits are more subtle, though *deepHTL* still displays discernible gains at n=2000. Importantly, we observe no performance penalty for the revision step. Despite the theoretical risk of added noise from the multi-step procedure, the method maintains accuracy in simpler settings, confirming the revision step as a safe and robust enhancement.

Overall, *deepHTL* outperforms all competing methods considered across the simulation grid. The largest margins occur at n=2000, confirming that the revision enables high capacity networks to leverage larger sample sizes effectively for resolving complex confounding and heterogeneity. These findings suggest a context aware strategy: while *deepHTL* excels in settings with complex confounding, large magnitude effects, and sufficiently large sample sizes, simpler learners (e.g., *R–KRR*) may suffice in smoother, lower-dimensional problems where computational efficiency is a priority. Returning to the clinical challenge that motivated this methodology, we apply *deepHTL* to a real-world lung transplant dataset in the following section.

## 4 A Lung Transplant Study

To demonstrate the practical utility of our proposed framework *deepHTL*, we apply it to registry data from the United Network for Organ Sharing (UNOS) to address a central question in lung transplantation: whether bilateral lung transplantation confers superior post transplant lung function measured by FEV1 compared with single lung transplantation, and whether any advantage varies across recipients and clinically relevant subgroups. Although BLT is often associated with improved lung function, the extent and clinical drivers of heterogeneity in its benefit remain underexplored (Oliveira et al., 2012; Force et al., 2011).

Our analytic cohort comprises 14,306 adult recipients transplanted between May 2005 and September 2011. The outcome Y is post transplant forced expiratory volume in 1 second (FEV1), a key marker of graft performance and long-term survival risk (Young et al., 2007). Treatment is encoded as Z∈{0,1}, with Z=1 indicating BLT (n=9,758) and Z=0 indicating SLT (n=4,548). The covariate vector X includes baseline donor and recipient characteristics used to adjust for confounding. To select adjustment variables in a data adaptive yet clinically grounded manner, we used the Group Lasso—Doubly Robust (GL-DR) procedure (Koch et al., 2018), which retained 26 clinically relevant predictors (e.g., recipient age, body mass index, and ischemic time) from 29 candidate covariates (Table 3).

We begin the analysis by formally testing for treatment effect heterogeneity using the revised null estimator. The analytical kernel score test is highly significant (p=0.002), and this finding is further corroborated by the permutation test (p=0.001). Together, these results provide strong evidence that the benefit of BLT varies across the recipient population. To further investigate the complexity of the detected heterogeneity, we estimate the effective degrees of freedom, $k_{\text{eff}}$, of the limiting mixture chi-squared distribution (Satterthwaite, 1946). Here, $k_{\text{eff}}$ acts as an interpretable proxy for the dimensionality of heterogeneity: values near 1 suggest a sparse pattern dominated by a single covariate direction, whereas larger values indicate a more diffuse and multidirectional structure. The estimated $k_{\text{eff}}\;\approx \;137.5$ is substantial, implying that the BLT-SLT contrast varies along many covariate directions and is driven by a complex interplay of recipient characteristics rather than a single dominant clinical factor.

**Figure 2:**
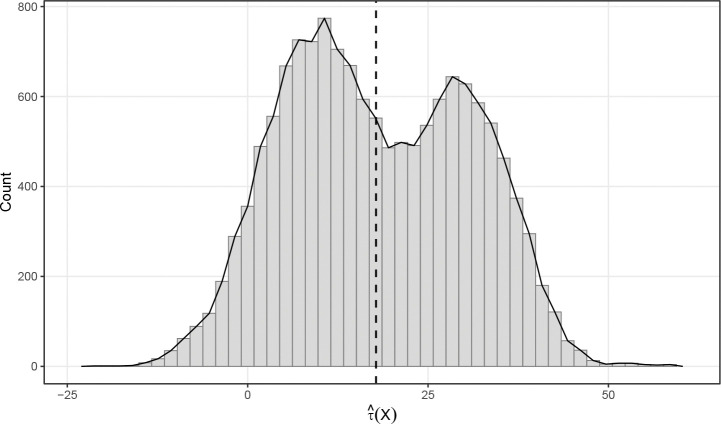
Estimated individualized treatment effects (BLT vs. SLT) in the whole UNOS lung transplant cohort. The dashed line denotes the average treatment effect.

Given the detected heterogeneity, we proceed to estimate τ(X), referred to hereafter as the individual treatment effects (ITE), using the *deepHTL* estimator. As a population level baseline, the estimated ATE is 17.79 (95% CI: 16.84,18.74) mL/sec, confirming that BLT confers a significant functional advantage on average. However, the distribution of estimated ITE (Figure 2) exposes substantial variation obscured by this aggregate metric. The effects span a wide range (*−*20.6 to 59.2 mL/sec) and exhibit a distinct bimodal structure. While the vast majority of patients (93%, n=13,210) are predicted to derive functional benefit from BLT, a clinically relevant minority (7%, n=986) are predicted to achieve superior outcomes with SLT. This suggests that a uniform BLT-for-all strategy risks exposing specific patient subgroups to the heightened surgical morbidity of bilateral transplantation without commensurate physiological gain.

**Figure 3:**
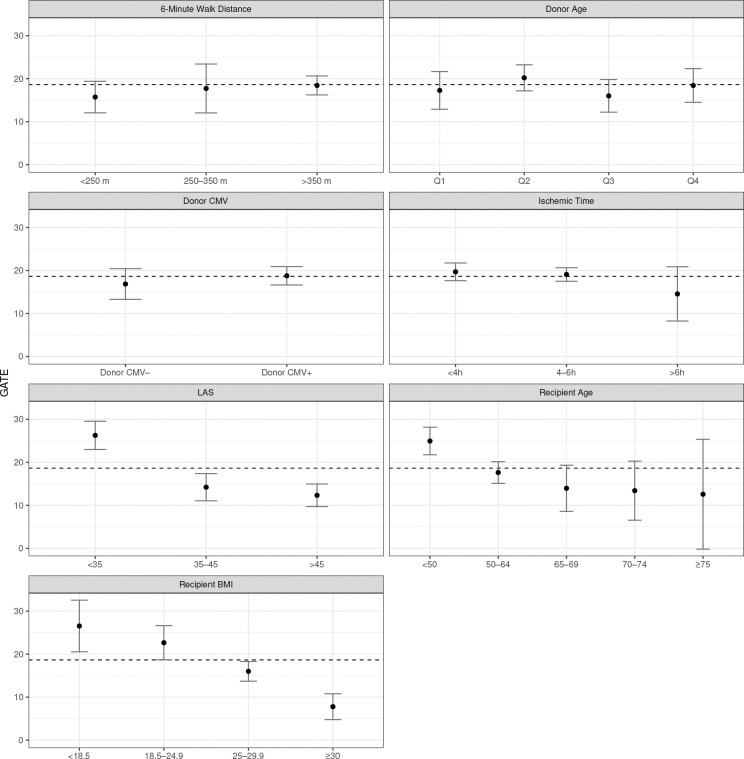
Estimation of GATE (BLT vs. SLT) across prespecified clinical subgroups. The horizontal dashed line denotes the average treatment effect.

To translate the individual estimates into actionable insights, we evaluate Group Average Treatment Effects (GATE) for key subpopulations (Figure 3). The functional advantage of BLT is the largest among patients with high physiological reserve, specifically, younger recipients (<50 years), those with low BMI (<18.5), and those with lower acuity (LAS < 35). Conversely, the benefit diminishes markedly for older recipients (≥75 years) and individuals with high metabolic burden (obese class II/III, BMI ≥ 30). Interestingly, donor characteristics such as ischemic time exert comparatively little influence on the differential benefit. These findings support a precision medicine approach to organ allocation: given the scarcity of donor organs, bilateral transplantation provides the greatest benefit among younger, low-risk, and lower-BMI candidates, whereas SLT remains a viable, resource-efficient option for older or higher-risk recipients. As a robustness check, an unsupervised k-means clustering (k=5) of the estimated ITE (see Figure S1) recovers clinically meaningful phenotypes that closely mirror these GATE patterns, confirming that the observed heterogeneity is driven by these core physiological characteristics.

Our findings should be interpreted in light of several limitations inherent to observational registry analyses. First, causal inference relies on the assumption of no unmeasured confounding. While we adjusted for 26 key clinical covariates selected via a robust GL-DR procedure, unrecorded factors such as donor’s lung quality, center-specific preferences for bilateral procedures, or granular frailty metrics guiding surgical candidate selection could simultaneously influence treatment assignment and FEV1 (Subramanian et al., 2018; Hayes et al., 2022). Second, we assume missing data were ignorable conditional on observed covariates, which may not hold for all variables. Finally, since the study cohort spans 2005 – 2011, generalizability to current practice may be affected by subsequent changes in donor allocation policies and surgical techniques. Despite these potential restrictions, the analysis demonstrates the utility of the proposed framework to uncover actionable heterogeneity in observational data.

## 5 Discussion

In this paper, we develop a unified framework for rigorous testing and estimation of heterogeneous treatment effects in observational studies with potentially complex confounding. Central to the framework is *deepHTL*, a revised deep semiparametric regression approach that targets both valid inference on the *existence* of heterogeneity and accurate estimation of covariate-dependent causal effects when heterogeneity is present. To gatekeep estimation with a statistically principled check, we propose a kernel score global test and a permutation test that achieve Type I error control under complex confounding structures regardless of the true homogeneous treatment effect magnitude, while maintaining robust power to detect potential complex heterogeneity. Extensive simulations demonstrate finite sample robustness, and an application to the UNOS lung transplant registry reveals clinically meaningful subgroups that drive differential functional benefit of bilateral versus single lung transplantation. Although motivated by a biomedical case study, the framework is broadly applicable to settings where effect variation must be disentangled from intricate confounding.

Methodologically, *deepHTL* refines and extends existing learners in several directions. Building on the quasi-oracle R-learner of (Nie and Wager, 2021), we introduce a revision step that stabilizes estimation of E(Y∣X) when treatment effects are not small, preventing the signal-to-noise–driven distortions that can invalidate downstream heterogeneity testing and the accuracy of HTE estimation. For nuisance function estimation, we use a bagged DNN ensemble (Mi et al., 2019, 2021; Zou et al., 2025) to reduce variance while retaining flexibility for complex (e.g., nonlinear and nonadditive) association. Finally, by embedding a global heterogeneity test as a first-stage decision rule, *deepHTL* addresses a critical but often overlooked inferential step and provides a rigorous procedure with controlled Type I error before estimating clinically meaningful individualized effects. It is worth pointing out that though *deepHTL* demonstrates its robustness for testing and estimating HTE under various confounding complexity, the validity relies on the assumption of absence of unmeasured confounding. Extending *deepHTL* to accommodate unmeasured confounding factors is practically important but it is out of scope of this paper.

## Figures and Tables

**Table 1:** Performance Comparison on Type I Error Control

τ	Estimator	n	p	σ	Type I Error (Anal.)	Type I Error (Perm.)

τ=0	Unrevised (${\hat{\tau }}_{0}$)	1000	20	1	0.042	0.049
		1000	20	3	0.056	0.052
		1000	40	1	0.062	0.066
		1000	40	3	0.083	0.061
		2000	20	1	0.045	0.048
		2000	20	3	0.058	0.059
		2000	40	1	0.055	0.061
		2000	40	3	0.060	0.066

	Revised (${\hat{\tau }}_{0}^{*}$)	1000	20	1	0.044	0.046
		1000	20	3	0.057	0.048
		1000	40	1	0.070	0.065
		1000	40	3	0.086	0.054
		2000	20	1	0.043	0.045
		2000	20	3	0.057	0.064
		2000	40	1	0.053	0.055
		2000	40	3	0.059	0.065

τ=3	Unrevised (${\hat{\tau }}_{0}$)	**1000**	**20**	**1**	**0.176**	**0.091**
		1000	20	3	0.064	0.049
		**1000**	**40**	**1**	**0.152**	**0.111**
		1000	40	3	0.086	0.064
		**2000**	**20**	**1**	**0.299**	**0.090**
		2000	20	3	0.072	0.058
		**2000**	**40**	**1**	**0.181**	**0.087**
		2000	40	3	0.070	0.063

	Revised (${\hat{\tau }}_{0}^{*}$)	1000	20	1	0.044	0.049
		1000	20	3	0.057	0.049
		1000	40	1	0.063	0.061
		1000	40	3	0.085	0.059
		2000	20	1	0.043	0.045
		2000	20	3	0.053	0.055
		2000	40	1	0.055	0.060
		2000	40	3	0.060	0.059

**Table 2:** Empirical Power for Detecting Complex HTE Using the Revised Estimator

n	p	σ	Power (Anal.)	Power (Perm.)

1000	20	1	0.998	1.000
1000	20	3	0.631	0.614
1000	40	1	0.953	0.940
1000	40	3	0.437	0.388

2000	20	1	1.000	1.000
2000	20	3	0.977	0.984
2000	40	1	1.000	1.000
2000	40	3	0.893	0.938

**Table 3:** Key patient and donor characteristics used as confounders by transplant type (BLT vs. SLT). The complete 26 covariates selected are shown in the Table S1.

Covariates	BLT	SLT
	(n = 9758)	(n = 4548)

Recipient Age (years), Mean (SD)	53.4 (13.5)	63.5 (7.5)
Recipient BMI, Mean (SD)	24.8 (4.7)	26.3 (4.1)
Lung Allocation Score, Mean (SD)	40.8 (13.5)	40.3 (11.1)
Diabetes, n (%)	1999 (20.5%)	836 (18.4%)
Recipient Height (cm), Mean (SD)	170.0 (9.9)	170.6 (9.6)
Ventilator Use, n (%)	465 (4.8%)	71 (1.6%)
6-Minute Walk Distance, Mean (SD)	773.8 (426.1)	794.6 (411.4)
Donor Age (years), Mean (SD)	34.7 (14.0)	34.9 (13.8)
Donor BMI, Mean (SD)	26.2 (5.6)	26.2 (5.3)
CMV Serology Positive, n (%)	6093 (62.4%)	2793 (61.4%)
Donor Diabetes, n (%)	701 (7.2%)	315 (7.0%)
Ischemic Time (hours), Mean (SD)	5.6 (1.76)	4.3 (1.52)

## Data Availability

The lung transplant registry data analyzed in this study were provided by the United Network for Organ Sharing (UNOS). Due to privacy restrictions and data use agreements, the raw clinical data cannot be made publicly available by the authors. Qualified researchers may request access to the data directly through the UNOS registry. The code used to implement the proposed methods and reproduce the simulation studies is available in the Supplementary Materials.
